# Notes on Preparations for the Sick

**Published:** 1911-06

**Authors:** 


### Notes on Preparations for the Sick.

**Turin International Exhibition, 1911.**—Many of our best manufacturing chemists and druggists occupy stands in the British Section of the Turin Exhibition, and medical men will find much to interest them in that portion of the building devoted to chemical and scientific industries. The exhibit of Burroughs, Wellcome & Co. is in particular very attractive.

Perhaps the most interesting novelty exhibited is a synthetic substance which possesses all the physiological characteristics of the supra-renal gland active principle. This product, which has been designated "Epinine," was discovered during researches in the firm's experimental laboratories at Dartford. After considerable clinical trial, it has proved so efficient that it has been placed on the market. Being purely a chemical substance of known composition, it is stable and is uniform in action.

Iodoform gauze was at one time considered to be unique in its own sphere, but a dressing is shown which is said to not only equal it in many ways, but even to surpass it. This new addition to the surgeon's armamentarium is known as "Tabloid" Bismuth Gauze. It is claimed that it excels iodoform gauze in every way, and especially for gynæcological work.

There are several other recent additions to medicine. Among these are "Lodal," a new synthetic substance valuable in uterine hemorrhage, and "Vaporole" Pituitary Extract, which has proved useful in cases of collapse.

A series of photographs of scenes from the "Wellcome" Materia Medica Farm which has been established in Kent is shown. These illustrate the scientific culture of herbs for use in the preparation of the firm's well-known products.

**Concentrated Diphtheria Antitoxin.**—BURROUGHS, WELLCOME & Co., London.—This preparation of Diphtheria Antitoxin has certain advantages over the ordinary diphtheria antitoxin serum. It is prepared at the Wellcome Physiological Research Laboratories according to the latest approved methods of salt-precipitation. On account of its concentration large doses can be given in comparatively small volume, a point of considerable importance, since large doses of antitoxin are the rule in the treatment of diphtheria, and the bulk of the serum is a factor which may militate against their being given. It is stated by some authorities that sera treated by the method used in preparing "Wellcome" Concentrated Diphtheria Antitoxin are less liable than the ordinary unconcentrated sera to produce rashes and other undesirable symptoms in susceptible patients. "Wellcome" Brand Concentrated Diphtheria Antitoxin is issued in hermetically-sealed phials of 1,000, 2,000, 3,000, 4,000 and 5,000 Ehrlich-Behring units, each 1,000 units being contained in 1 c.c. or less of fluid.

---

**Soloid Nizin.**—BURROUGHS, WELLCOME & Co., London.—"Nizin," a zinc salt of sulphanilic acid, is a valuable antiseptic, readily soluble in water, and in solutions of the strengths recommended produces neither irritation nor toxic effects.

Clinical reports prove the great value of "Nizin" in gonorrhœa. It can be injected safely in the acute inflammatory stage. Urethral and vaginal injections of solutions of a strength of 2 to 6 grains to the ounce have proved very successful. A solution of a similar strength forms an excellent stimulating antiseptic application for use in indolent ulcers. Weaker solutions are employed in gonorrhœal ophthalmia, conjunctivitis and other eye affections.

"Soloid Nizin" affords a ready and convenient means of preparing fresh solutions for injections and eye lotions in the strengths of gr. 2 and gr. 20, the former in bottles of 100 and the latter in bottles of 25.

---

**Chapman's "Entire Wheat" Food.**—CHAPMAN'S FOOD CO., Christchurch, Hants.—This preparation is intended for the use of children of seven months and upwards and for invalids. An analysis shows it to be a farinaceous food, containing proteid matter in considerable amount. An examination of the inorganic residue left after burning off the organic matter revealed the presence of phosphates in considerable amount. An excellent feature of the food is the absence of added sweetening matter, which so often spoils otherwise desirable preparations.

**Chapman's "Malted Food."**—This preparation differs from the "Entire Wheat" Food in containing malted barley, and is thus suitable for younger children who are unable to digest the ordinary starch-containing foods. Both this and the unmalted food can be recommended as satisfactory foods at a moderate price.

---

**Standard Wholemeal Biscuits.**—MACFARLANE, LANG & CO., Fulham, London.—The name Standard appears now to be looked upon as the acme of excellence, and these biscuits, manufactured from stone-ground flour and containing all the food value of the whole wheat, are suitably sweetened, crisp and palatable. They are certainly good and agreeable ; but we fail to see why bread and biscuit eaters should be limited to one variety of either, as seems to be desired by the puffing advertisements of wholemeal bread.

---

**Pulverettes and Palatinoids.**—OPPENHEIMER, SON & CO. LTD., 179 Queen Victoria Street, London. — **Calcium Iodide Pulverettes.**—This drug is nauseous in taste, and deteriorates rapidly when exposed to the air. In pulverette form it is protected from the atmosphere, and may be swallowed tastelessly. In doses of 5 to 15 grains they have recently given excellent results in cases of diabetes, sometimes when dieting and Codeine treatment have failed. They are free from sugar.

**Calcium Permanganate,** gr. $\frac{1}{4}$.—This salt is very deliquescent, and has an acrid, nauseous taste. The hermetically-sealed jujube surmounts both difficulties, and the contents being in a loose condition diluted with an inert powder are readily absorbed.

**Iodsam** gr. 3 (25 per cent. Iodine).—This pulverette contains the iodine in a peppermint-flavoured oil basis, and is equivalent to one grain of Potassium Iodide.

**Filmaron** (the active principle of male fern) Palatinoid.—Three of these taken fasting and followed by a dose of castor oil one hour later are an efficient dose for the expulsion of tapeworm. They have none of the objections of the ordinary preparation of male fern.

---

**"Aseptule" Sea-water Plasma.**—OPPENHEIMER, SON & CO. LTD., 179 Queen Victoria Street, London.—In the *Medical Annual*, 1910, Dr. Robert Simon, Paris, published some remarkable results which he obtained after extended trial with subcutaneous injection of diluted sea-water.

It proved to be an exceedingly powerful tonic and alterative, restoring appetite, digestion and sleep, correcting old standing neuroses, promoting healing of wounds, improving tuberculous esions, checking marasmus and gastro-entritis in infants, and exerting a powerful curative influence on various skin diseases.

It is said to exercise an absolute, rapid and radical effect on the following diseases:—(1) acute gastro-intestinal affections of infancy, (2) digestive disorders of adults and the resulting neuroses, (3) skin diseases.

Messrs. Oppenheimer have completed arrangements whereby the sea-water is collected at frequent intervals 200 miles from land (in the deep North Sea) under strictly aseptic conditions. It is sterilised by filtration through a porcelain filter, and rendered isotonic with the blood; it is issued in Aseptules of 30, 50 and 100 c.c. capacity.

We fail to see on what grounds diluted sea-water should be glorified by the designation Plasma.

